# The epigenetic trajectory of type 1 regulatory T cells

**DOI:** 10.7554/eLife.104239

**Published:** 2024-11-14

**Authors:** David P Turicek, Xiaoxiao Wan

**Affiliations:** 1 Division of Immunobiology, Department of Pathology and Immunology, Washington University School of Medicine St Louis United States; 2 The Andrew M. and Jane M. Bursky Center for Human Immunology and Immunotherapy Programs, Washington University School of Medicine St Louis United States

**Keywords:** epigenetics, immunoregulation, T follicular helper cells, type 1 regulatory T cells, transdifferentiation, nanomedicine, Mouse

## Abstract

The epigenome of T follicular helper cells prepares them for conversion into type 1 regulatory T cells.

**Related research article** Garnica J, Solé P, Yamanouchi J, Moro J, Mondal D, Fándos C, Serra P, Santamaria P. 2024. Garnica J, Solé P, Yamanouchi J, Moro J, Mondal D, Fándos C, Serra P, Santamaria P. 2024. T-follicular helper cells are epigenetically poised to transdifferentiate into T-regulatory type-1 cells. *eLife* **12**:RP97665. doi: 10.7554/eLife.97665.

Autoimmunity – when the immune system attacks healthy tissues in an individual – is often due to the failure of regulatory mechanisms to suppress immune cells that react to healthy tissue, which are known as ‘autoreactive’. Historically, steroids have been used to treat autoimmunity by dampening the activity of the entire immune system. However, this systemic suppression makes individuals more susceptible to infection. More recently, immunotherapies have been designed to target specific cell types or signaling pathways. While these therapeutics can block autoreactive cells, they often still block non-autoreactive cells too. A better understanding of how immune cells are regulated could help to develop therapies that specifically target autoreactive cells.

Several cell types are involved in suppressing immune cell activity, with perhaps the most characterized being regulatory T cells (or Tregs for short). These cells can suppress other immune cells indirectly by producing cytokines and directly through interacting with co-stimulatory molecules on the surface of cells that initiate immune responses. Less is known about a distinct subset of Tregs called type 1 regulatory T cells (known as TR1 cells for short) and their role in suppressing immune responses (Roncarolo et al., 2018). Now, in eLife, Pere Santamaria and colleagues from the Institut D’Investigacions Biomèdiques August Pi I Sunyer and The University of Calgary – including Josep Garnica as first author – report insights into the mechanisms regulating the gene activity required for TR1 cells to develop (Garnica et al., 2024).

Previous work from the same laboratory showed that T follicular helper (TFH) cells – which are involved in helping B cells generate antibodies – can be re-programmed into TR1 cells when exposed to nanoparticles coated with disease-specific peptides. These nanoparticles function as a delivery system, presenting the peptides to TFH cells as an approach for treating autoimmunity (Clemente-Casares et al., 2016). The resulting TR1 cells reversed autoimmunity without impairing normal functioning of the immune system (Singha et al., 2017; Umeshappa et al., 2019; Solé et al., 2023a). A key transcription factor, known as BLIMP-1 was identified as the master regulator of this conversion process (Solé et al., 2023b).

Building on this previous work, Garnica et al. employed several tools to examine how the control of gene activity, or ‘epigenetics’, affects the re-programming of TFH cells into TR1 cells. The accessibility of chromatin – a complex of DNA and proteins that compacts DNA into chromosomes – can influence the likelihood of gene expression. Analyzing chromatin accessibility in each of the cell types showed that several chromatin regions close during conversion, while several others open. Furthermore, thousands of genes were expressed differently between TFH and TR1 cells. Indeed, the upregulated genes corresponded to regions of chromatin that remained accessible, while the downregulated genes corresponded to chromatin regions that closed during the conversion process.

Garnica et al. next looked at post-translational changes to DNA and the histone proteins that bind to it to see if they also affect gene expression during TFH to TR1 cell conversion (Figure 1). For the genes upregulated in TR1 cells but not expressed in TFH cells (despite having accessible chromatin), their analysis revealed that these genes already had their respective histone marks at the TFH stage. Another way to control gene expression is to modify DNA by adding a methyl group via a process called methylation, which tends to make genes less likely to be expressed. Similarly to the histone markers, the methylation status of TR1 cells was already present at the TFH cell stage, suggesting that changes in gene expression during conversion are not due to DNA methylation changes.

**Figure 1.**
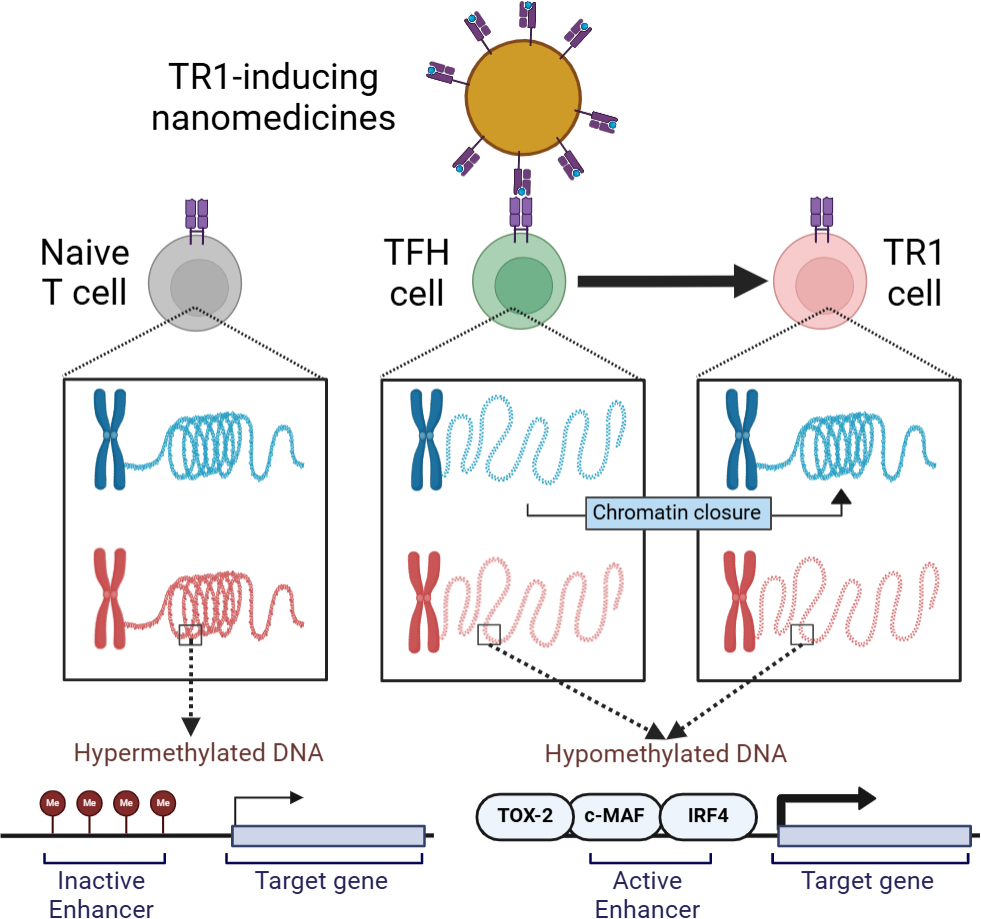
How epigenetics drives the conversion of T follicular helper cells into immunosuppressive Type 1 regulatory T cells. TR1-relevant chromatin regions (blue) are inaccessible in naïve T cells (gray; left) and the DNA at relevant sites is highly methylated in order to keep them inactive. On the other hand, the same sites in TFH cells (green; middle) become active through demethylation. This ensures TFH cells are poised to be converted into TR1 cells (pink) when TR1-inducing nanomedicines (yellow; top) activate them through self-surface molecules (blue and pink structures). This leads to chromatin closure in certain regions and expression of active enhancer-containing genes through transcription factors such as TOX-2, c-MAF, and IRF4. TR1: T-regulatory type-1 cell, TFH cell: T-follicular helper cell.

This figure was created with BioRender.com.

These findings prompted Garnica et al. to hypothesize that the conversion process is fueled by changes in the expression and binding of transcription factors that either stabilize TFH cells or promote TR1 cells. Intriguingly, losing TFH-specific transcription factor gene expression during conversion was associated with chromatin closure. For the relevant chromatin regions that remain open during the conversion process, TR1 cells inherited stretches of DNA, called enhancers, from their TFH precursors, which increase gene expression. Whereas in naïve T cells these genes were highly methylated and inaccessible, in TFH and TR1 cells they were less methylated and instead were enriched in binding sites for TFH-transcription factors such as TOX-2, IRF4, and c-MAF. Taken together, the findings suggest that the TR1 transcriptional program is genetically imprinted at the TFH cell stage.

While Garnica et al. have shown the ability of TR1 cells to reverse autoimmunity, TR1 cells have also been shown to hinder some cancer immunotherapies (Sultan et al., 2024). TR1 cells that developed in response to high doses of antigen impaired tumor rejection, even when immune checkpoint therapy was used to boost the immune response against the cancer cells. Therefore, TR1 cells are a double-edged sword in preventing undesirable autoimmunity and simultaneously suppressing desirable immune responses.

Therapeutically, one could imagine harnessing the beneficial anti-autoimmune effects of TR1 cells in an antigen-specific manner while avoiding their detrimental pro-cancer activity. However, doing so requires a thorough understanding of TR1 development and function in both homeostasis and disease. The findings of Garnica et al. provide the first robust examination of the epigenetic changes that occur during TFH-to-TR1 conversion. Current research by the same team is ongoing to examine how different transcription factors affect the transcriptional and chromatin changes during TFH-TR1 conversion.
